# Loss of parkin promotes lipid rafts-dependent endocytosis through accumulating caveolin-1: implications for Parkinson’s disease

**DOI:** 10.1186/s13024-015-0060-5

**Published:** 2015-12-01

**Authors:** Seon-Heui Cha, Yu Ree Choi, Cheol-Ho Heo, Seo-Jun Kang, Eun-Hye Joe, Ilo Jou, Hwan-Myung Kim, Sang Myun Park

**Affiliations:** Department of Pharmacology, Ajou University School of Medicine, 164, Worldcup-ro, Yeongtong-gu, Suwon, 16499 Korea; Chronic Inflammatory Disease Research Center, Ajou University School of Medicine, Suwon, Korea; Neuroscience Graduate Program, Ajou University School of Medicine, Suwon, Korea; Department of Chemistry, Ajou University, Suwon, Korea

**Keywords:** Parkin, Caveolin-1, Endocytosis, Cell-to-cell transmission

## Abstract

**Background:**

Parkinson’s disease (PD) is characterized by progressive loss of midbrain dopaminergic neurons, resulting in motor dysfunctions. While most PD is sporadic in nature, a significant subset can be linked to either autosomal dominant or recessive mutations. *PARK2*, encoding the E3 ubiquitin ligase, parkin, is the most frequently mutated gene in autosomal recessive early onset PD. It has recently been reported that PD-associated gene products such as PINK1, α-synuclein, LRRK2, and DJ-1, as well as parkin associate with lipid rafts, suggesting that the dysfunction of these proteins in lipid rafts may be a causal factor of PD. Therefore here, we examined the relationship between lipid rafts-related proteins and parkin.

**Results:**

We identified caveolin-1 (cav-1), which is one of the major constituents of lipid rafts at the plasma membrane, as a substrate of parkin. Loss of parkin function was found to disrupt the ubiquitination and degradation of cav-1, resulting in elevated cav-1 protein level in cells. Moreover, the total cholesterol level and membrane fluidity was altered by parkin deficiency, causing dysregulation of lipid rafts-dependent endocytosis. Further, cell-to-cell transmission of α-synuclein was facilitated by parkin deficiency.

**Conclusions:**

Our results demonstrate that alterations in lipid rafts by the loss of parkin via cav-1 may be a causal factor of PD, and cav-1 may be a novel therapeutic target for PD.

## Background

Parkinson’s disease (PD) is a common neurodegenerative disease characterized by progressive degeneration of midbrain dopaminergic neurons, resulting in motor dysfunction [1]. Although the majority of PD occurs sporadically, mutations in several genes including *SNCA, LRRK2, parkin, PINK1,* and *DJ-1* have been identified in patients with familial PD, providing enormous insight into the molecular pathways underlying the neurodegeneration shown in PD [2]. Despite the fact that it has not yet been fully unveiled, oxidative stress, mitochondrial damage and defects of protein quality control system are known to play a critical role in the pathogenesis of PD [1]. Recently, prion-like propagation of α-synuclein, encoded by *SNCA*, a PD-associated gene, has also been proposed to play a role in the progression of PD [3–5].

Parkin is a 465 amino acid protein that possesses E3 ubiquitin ligase activity, and familial type mutations in parkin are predicted to be loss of function [6], suggesting that loss of parkin activity results in the gradual and abnormal accumulation of parkin substrates, which may cause familial PD. Mutations in the parkin gene have been revealed to be the most common cause of autosomal recessive early onset PD, which causes 50 % of autosomal recessive early onset PD [7]. In addition, increasing evidence indicates that parkin is also inactivated in patients with sporadic PD by s-nitrosylation, tyrosine phosphorylation, and oxidation [8], suggesting that parkin also plays a critical role in the pathogenesis of sporadic PD. Accordingly, the identification of parkin substrates is important to elucidate the exact roles of parkin in the pathogenesis of PD. To date, several substrates including CDCrel-1 [9], Pael-R [10], synphilin-1 [11], and PARIS [12] have been identified and great efforts is continuously being made in the identification of novel substrates to elucidate the pathogenesis of PD.

Parkin is localized mainly in the cytosol, however, a small proportion has been shown to be localized in membranes including in lipid rafts [13–15]. Lipid rafts are specialized membrane microdomains that are enriched in cholesterol, glycosphingolipids, and glycosylphosphatidylinositol (GPI)-anchored proteins, which serve as organization centers for the assembly of signaling molecules, and influence membrane fluidity and trafficking of membrane proteins, and regulate receptor-mediated signal transduction and exo/endocytosis [16, 17]. Caveolins and flotillins have been shown to be integral components of lipid rafts [18]. Recently, increasing evidence has indicated that lipid rafts may play an important role in neurodegeneration [19, 20]. In particular, many PD-associated gene products such as α-synuclein [21], PINK1 [22], LRRK2 [23], and DJ-1 [24], as well as parkin [13], have also been reported to associate with lipid rafts, implying that functional alteration of lipid rafts by these proteins may be one of the common pathological mechanisms of PD. We recently reported that DJ-1 regulates lipid rafts-dependent endocytosis [24], however, the roles of other proteins in lipid rafts have yet to be explored.

In the present study, we explore whether parkin regulates the components of lipid rafts and whether loss of parkin induces functional alterations in lipid rafts. In addition, we explore the association between the functional alterations in lipid rafts induced by loss of parkin and the propagation of α-synuclein, which has been recently proposed to be a causal factor of the progression of PD.

## Results

### Loss of parkin leads to an increase in caveolin-1 (cav-1)

**Fig. 1:**
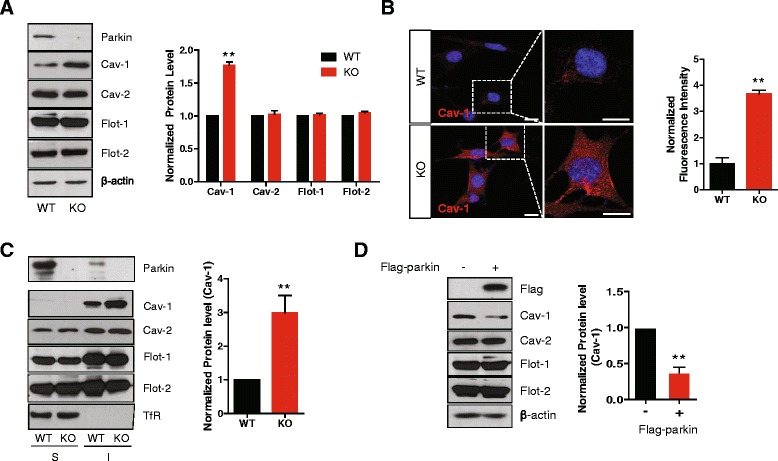
Loss of parkin leads to an increase in cav-1. **a** Lysates prepared from WT and parkin KO MEF cells were analyzed by SDS-PAGE and Western blotting, and the band intensity of three independent experiments was quantified. **b** WT and parkin KO MEF cells were stained with anti-caveolin-1 (red) and observed by confocal microscopy. The intensity of three independent experiments was quantified. The mean intensity value of each experiment was acquired by measuring the intensity of at least 100 cells. Scale bar indicates 10 μm. **c** WT and parkin KO MEF cells were lysed in ice-cold 1 % Triton X-100 buffer and fractionated as described in ‘Methods’. The soluble and insoluble fractions were then analyzed using Western blotting. The transferrin receptor (TfR) was used as a marker for the non-lipid raft fractions. Band intensity of three independent experiments was quantified. **d** Parkin KO MEF cells were transfected with a plasmid for flag-parkin, and after 48 h, lysates were analyzed by SDS-PAGE and Western blotting, and the band intensity of three independent experiments was quantified. *P* values were determined using a Student’s *t* test. ** *p* < 0.01

To explore whether loss of parkin induces functional alterations in lipid rafts, we first compared the expression level of the well-known lipid rafts marker proteins, caveolin-1 (cav-1), cav-2, flotillin-1 (flot-1), and flot-2 using WT and parkin KO MEF cells. Interestingly, we observed that the level of cav-1 was increased in parkin KO MEF cells specifically, while the levels of other proteins were not changed (Fig. 1a). Confocal microscopic analysis also shows an increase of cav-1 protein in parkin KO MEF cells compared with WT MEF cells (Fig. 1b). To investigate the distribution of increased cav-1 in parkin KO MEF cells, lipid rafts were isolated based on their solubility in 1 % Triton X-100 on ice [24, 25]. As shown in Fig. 1c, the increase in cav-1 protein in parkin KO MEF cells was observed only in the cold Triton X-100 insoluble fraction. Moreover, the distribution of the other proteins was not changed. The expression of parkin was relatively lower in MEF cells than that in neurons. Therefore, we loaded a 4-fold higher amount of protein for Western blotting of parkin and a small proportion of parkin was detected in the cold Triton X-100 insoluble fraction. This is also in agreement with a previous study [13]. To test whether parkin regulates cav-1 expression specifically, we overexpressed flag-parkin in parkin KO MEF cells. The overexpression of parkin in parkin KO MEF cells rescued cav-1 level without any change in the levels of other proteins (Fig. 1d), suggesting that parkin regulates cav-1 specifically.

### Parkin induces the degradation of cav-1 through the proteasome-dependent pathway

**Fig. 2:**
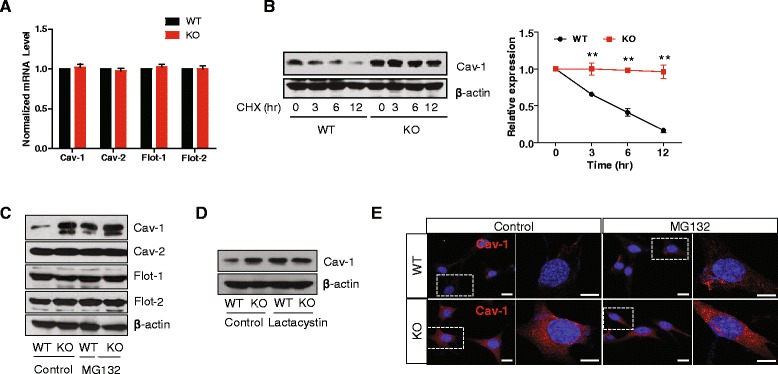
Parkin induces the degradation of cav-1 through the proteasome-dependent pathway. **a** Real-time RT-PCR was performed as described in ‘Methods’. WT and parkin KO MEF cells were incubated with with 10 μg/ml cyclohexamide (CHX) for the indicated times **b** 10 μM MG132 for 3.5 h **c** and 10 μM lactacystin for 3.5 h **d** Lysates were analyzed by SDS-PAGE and Western blotting. *P* values were determined using a two way ANOVA, **p < 0.01. **e** WT and parkin KO MEF cells cultured on poly-D-lysine coated glass were incubated with 10 μM MG132 for 3.5 h, stained with anti-cav-1 (red), and then observed by confocal microscopy. Blue indicates DAPI staining. Scale bar indicates 10 μm

**Fig. 3:**
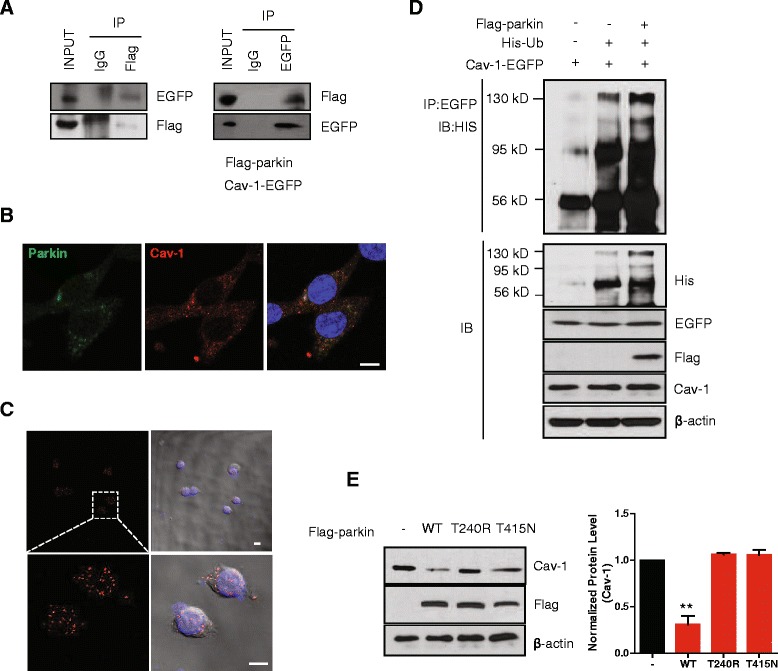
Parkin interacts with and ubiquitinates cav-1. **a** Parkin KO MEF cells were transfected with plasmids for flag-parkin and cav-1-EGFP for 48 h, and then the lysates were immunoprecipitated with anti-flag and anti-EGFP antibodies, respectively, followed by Western blotting. **b** WT MEF cells cultured on poly-D-lysine-coated glass were co-stained with anti-cav-1 (red) and anti-parkin (green), and observed by confocal microscopy. Blue indicates DAPI staining. Scale bar indicates 10 μm. **c** An *in situ* PLA assay was performed in WT MEF cells, as described in ‘Methods’. Red PLA spots represent interactions between parkin and cav-1. Blue indicates DAPI staining. Scale bar indicates 10 μm. **d** Parkin KO MEF cells were transfected with the indicated combinations of plasmids for flag-parkin, His-ubiquitin (His-Ub), and cav-1-EGFP for 48 h, and were then incubated with 10 μM MG132 for 3.5 h. The lysates were immunoprecipitated with an anti-EGFP antibody, followed by Western blotting. **e** Parkin KO MEF cells were transfected with plasmids for flag-tagged WT parkin and mutants (T240R and T415N) for 48 h, and the lysates were then analyzed by SDS-PAGE and Western blotting. The band intensity of three independent experiments was quantified. *P* values were determined using one way ANOVA. ** *p* < 0.01

To investigate in more detail the manner in which the loss of parkin leads to an increase in cav-1, we tested the mRNA level of cav-1 in both WT and parkin KO MEF cells. Quantitative RT-PCR shows that the mRNA level of cav-1, as well as that of cav-2, flot-1, and flot-2 were not different between the two cell lines (Fig. 2a), suggesting that parkin does not regulate cav-1 at the transcriptional level. Parkin regulates protein degradation as an E3 ubiquitin ligase [6]. In addition, cav-1 has been reported to be partially degraded by the ubiquitin proteasome system (UPS) [26, 27], speculating that parkin may regulate cav-1 degradation via the UPS. To confirm this hypothesis, WT and parkin KO MEF cells were treated with cycloheximide (CHX), an inhibitor of protein synthesis, and the expression of cav-1 was assessed using Western blotting. As shown in Fig. 2b, the degradation of cav-1 in WT MEF cells was significantly faster than in parkin KO MEF cells. In addition, while treatment with the proteasomal inhibitors, MG-132 and lactacystin, led to an accumulation of cav-1 in WT MEF cells, it did not further enhance the steady state levels of cav-1 in parkin KO MEF cells. (Fig. 2c and d). Confocal microscopic analysis confirmed this observation (Fig. 2e), suggesting that parkin regulates cav-1 degradation via the UPS. These results also prompted speculation that cav-1 may be a substrate of parkin, thus, we tested the interaction between the two proteins. As shown in Fig. 3a, both flag-parkin and cav-1-EGFP were overexpressed in parkin KO MEF cells, and immunoprecipitation shows their interaction. Antibody staining of endogenous parkin and cav-1 in MEF cells shows the colocalization of both proteins (Fig. 3b), which was also confirmed using an *in situ* proximity ligation assay (PLA) (Fig. 3c), suggesting that parkin interacts with cav-1. Next, in order to investigate whether parkin ubiquitinates cav-1, we transfected parkin KO MEF cells with flag-parkin, His-ubiquitin, and cav-1-EGFP, which resulted in an increase in the ubiquitination of cav-1-EGFP by flag-parkin (Fig. 3d). Furthermore, mutants of parkin that have been identified in patients with familial PD did not rescue the increase in cav-1 by loss of parkin (Fig. 3e), suggesting that parkin mediates the ubiquitination of cav-1, thereby targeting cav-1 to the proteasome for degradation.

### Cholesterol level, membrane fluidity, and lipid rafts-dependent endocytosis were altered in parkin KO MEF cells

**Fig. 4:**
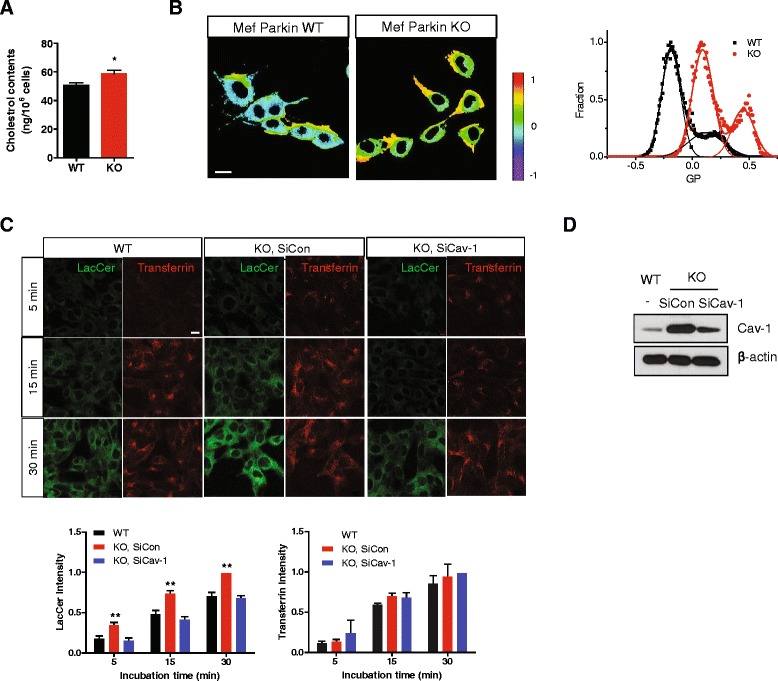
Cholesterol level, membrane fluidity, and lipid rafts-dependent endocytosis are altered in parkin KO MEF cells. **a** The total cholesterol level was measured in WT and parkin KO MEF cells, as described in ‘Methods’. *P* values were determined using a Student’s t-test. *p < 0.05. **b** WT and parkin KO MEF cells were stained with 0.5 μM C-laurdan, and then observed by two-photon microscopy. Images were processed to obtain GP values as described in ‘Methods’. Scale bar indicates 10 μm. **c** WT and KO MEF cells transfected with siRNA for non-targeting (siCon) and cav-1 (siCav-1) for 48 h, were incubated with 50 nM BOIPY® FL C_5_-Lactosylceramide and 2.5 μg/ml rhodamine-conjugated transferrin for the indicated times. The cells were then fixed and observed by confocal microscopy. Scale bar indicates 20 μm. The intensity of three independent experiments was quantified. The mean value of intensity of each experiment was acquired by measuring the intensity of at least five random fields. *P* values were determined using a Two way ANOVA. **p < 0.01. **d** Parkin KO MEF cells were transfected with siRNA for non-targeting (SiCon) and cav-1 (SiCav-1) for 48 h, and then the lysates were analyzed by SDS-PAGE and Western blotting

Cav-1 is known to regulate cholesterol transport [28, 29]. Accordingly, we measured the total cellular cholesterol level. As shown in Fig. 4a, the total cholesterol level in parkin KO MEF cells was slightly increased compared with that in WT MEF cells. Upon measurement of membrane fluidity using C-laurdan [30], the calculated GP value in parkin KO MEF cells was higher than that in WT MEF cells, suggesting that the membrane fluidity was decreased (Fig. 4b). This indicates that loss of parkin leads to an alteration in the total cholesterol level and membrane fluidity. Cav-1 has also been shown to regulate lipid rafts-dependent endocytosis [31]. In addition, the cholesterol level influences membrane fluidity, which can affect endocytosis [32]. Accordingly, in order to investigate whether parkin regulates lipid rafts-dependent endocytosis via cav-1, we performed an *in vitro* endocytosis assay using LacCer as a marker of lipid rafts-dependent endocytosis [31, 33] and transferrin as a marker of clathrin-dependent endocytosis [34, 35]. As shown in Fig. 4c, the level of endocytosis of LacCer was higher in parkin KO MEF cells than in WT MEF cells, however, endocytosis of transferrin was not different between the two cell lines. Furthermore, to confirm that the increased level of endocytosis of LacCer in parkin KO MEF cells was due to the accumulation of cav-1, we downregulated cav-1 using siRNA. Transfection of cav-1 siRNA efficiently downregulated cav-1 expression (Fig. 4d), and endocytosis of LacCer in parkin KO MEF cells was rescued by the downregulation of cav-1 expression (Fig. 4c), suggesting that parkin regulates lipid rafts-dependent endocytosis via cav-1.

### Parkin regulates lipid rafts-dependent endocytosis via cav-1 in neurons

**Fig. 5:**
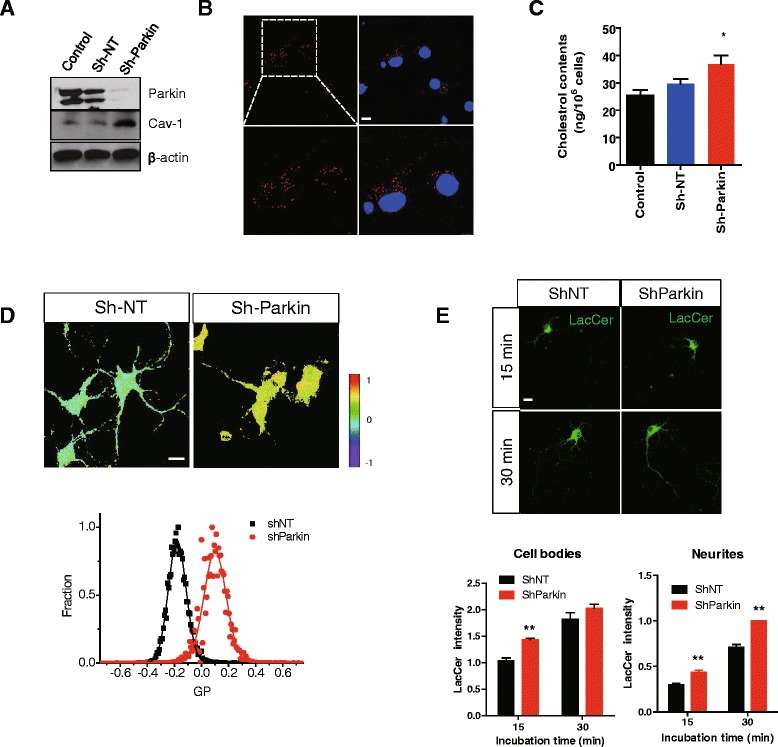
Parkin regulates lipid rafts-dependent endocytosis via cav-1 in neurons. **a** Rat primary cortical neurons were infected with shRNA lentivirus for non-targeting (Sh-NT) and parkin (Sh-Parkin) 11 days after plating, and cultured for a further 3 days. The lysates were analyzed by SDS-PAGE and Western blotting. **b** An *in situ* PLA assay was performed in rat primary cortical neurons, as described in ‘Methods’. Red PLA spots represent interactions between parkin and cav-1. Blue indicates DAPI staining. Scale bar indicates 20 μm. **c** The total cholesterol level was measured, as described in ‘Methods’. *P* values were determined using One way ANOVA. *p < 0.05. **d** Rat primary cortical neurons were infected with shRNA lentivirus for non-targeting (Sh-NT) and parkin (Sh-Parkin), stained with 0.5 μM C-laurdan, and then observed by two-photon microscopy. Images were processed for obtaining GP values as described in ‘Methods’. Scale bar indicates 20 μm. **e** Rat primary cortical neurons were infected with shRNA lentivirus for non-targeting (Sh-NT) and parkin (Sh-Parkin) and incubated with 50 nM BOIPY® FL C_5_-Lactosylceramide The cells were then fixed and observed by confocal microscopy. Scale bar indicates 20 μm. The intensity of three independent experiments was quantified. The mean value of intensity of each experiment was acquired by measuring the intensity of at least five random fields. *P* values were determined using a Two way ANOVA. **p < 0.01

We performed all the experiments in MEF cells, thus, in order to confirm whether parkin regulates cav-1 in neurons, we suppressed parkin expression in primary cortical neurons using an shRNA lentiviral system. As shown in Fig. 5a, parkin level was efficiently suppressed in neuron by the shParkin lentivirus, and as a result, cav-1 expression was increased, suggesting that parkin regulates cav-1 in neurons. In addition, an *in situ* PLA was performed in primary cortical neurons, and the interaction of parkin with cav-1 was observed (Fig. 5b). The total cholesterol level was increased (Fig. 5c), and the membrane fluidity of parkin KD neurons was decreased compared with that of WT neurons (Fig. 5d). Moreover, the endocytosis of LacCer was enhanced by parkin knockdown (Fig. 5e), which is in agreement with our data in MEF cells (Fig. 4), suggesting that parkin regulates lipid rafts-dependent endocytosis via cav-1 in neurons.

### Loss of parkin facilitates the transmission of α-synuclein

**Fig. 6:**
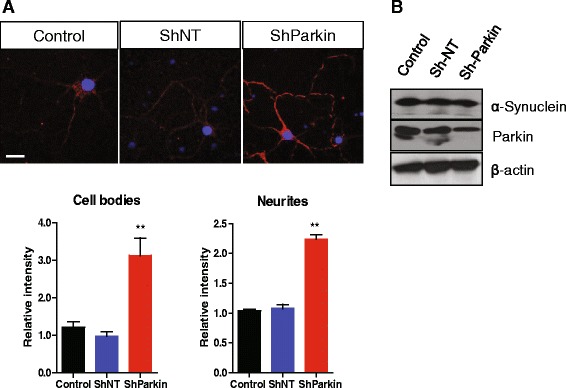
Loss of parkin facilitates the transmission of α-synuclein. **a** Rat primary cortical neurons were infected with shRNA lentivirus for non-targeting (Sh-NT) and parkin (Sh-Parkin) 11 days after plating, cultured for a further 2 days, and then co-cultured with α-synuclein overexpressing SH-SY5Y cells for 24 h. The cells were stained with an anti-human α-synuclein (red) antibody and observed using confocal microscopy. Blue indicates DAPI staining. Scale bar indicates 20 μm. **b** Rat primary cortical neurons were infected with shRNA lentivirus for non-targeting (Sh-NT) and parkin (Sh-Parkin) 11 days after plating and cultured for a further 3 days. The lysates were analyzed by SDS-PAGE and Western blotting

Previously, we have demonstrated that α-synuclein is internalized by lipid rafts-dependent endocytosis in BV-2 cells, a murine microglial cell line [36]. To investigate whether parkin regulates the internalization of α-synuclein into neurons, we used the two chamber culture system using human α-synuclein overexpressing SH-SY5Y cells, which showed the efficient internalization of cell-released α-synuclein into neighboring cells [36, 37]. Following staining with α-synuclein using a human α-synuclein-specific antibody in a co-culture of α-synuclein overexpressing SH-SY5Y cells and rat primary cortical neurons, cell-derived α-synuclein was observed to be internalized in much higher amounts into rat primary cortical neurons suppressing parkin expression than into control neurons (Fig. 6a), suggesting that loss of parkin could regulate the propagation of α-synuclein. To rule out the possibility that the higher α-synuclein intensity in rat primary cortical neurons suppressing parkin expression was due to abnormal degradation of α-synuclein by dysfunction of parkin, we determined the endogenous α-synuclein level following suppression of parkin expression in primary neurons. The level of endogenous α-synuclein was not different between the primary cortical neurons infected with parkin (shParkin) and with non-targeting shRNA lentivirus (shNT) (Fig. 6b), suggesting that parkin regulates α-synuclein transmission rather than degradation.

## Discussion

Increasing evidence indicates that alteration in lipid rafts is observed in many neurodegenerative diseases. Alterations in lipid composition have been reported in lipid rafts from AD and PD human brain cortex [38, 39]. Alterations in lipid rafts proteins have also been observed in AD and amyotrophic lateral sclerosis mouse models [40, 41]. Based on these observations and data from several studies, altered lipid rafts organization has also been proposed to be a common causative factor of many neurodegenerative diseases [19, 20].

In the present study, we identified cav-1 as a novel substrate of parkin. Cav-1 is a main structural protein component of caveolae, a subset of lipid rafts [42]. We observed that among the main structural protein components of lipid rafts, cav-1 is specifically accumulated in parkin KO MEF cells and primary neurons suppressing parkin expression. We also demonstrated that WT parkin ubiquitinates cav-1 for degradation. The N-terminal region of cav-1 has been reported to be exclusively ubiquitinated [27], suggesting that parkin can ubiquitinate any of the N-terminal lysine residues of cav-1. On the contrary, mutations in parkin, which are associated with familial PD, failed to regulate cav-1 expression, suggesting that accumulation of cav-1 may be involved in the pathogenesis of PD. Cav-1 is a 21-24 kDa membrane protein, which is a member of the caveolin family consisting of cav-1, 2, and 3. Caveolins have diverse functions including membrane-initiated intracellular signaling via the clustering of proteins, the segregation of proteins, and the trafficking of proteins to and from the membrane and lipid transport [43, 44]. They are also known to be involved in diverse pathologies including carcinogenesis, insulin resistance, and aging [45]. Although the roles of cav-1 in the CNS have been relatively less studied, an association between cav-1 and neurodegeneration and aging has been reported. Cav-1 expression levels in the hippocampus are upregulated in AD compared with control brains [46]. An age-related increase in cav-1 has also been reported [47–49]. On the contrary, loss of cav-1 in young mice has been reported to accelerate neurodegeneration and aging [50, 51]. Although the relationship between the level of cav-1 and neurodegeneration is controversial, cav-1 may be a critical control point for cellular aging [52]. Given that aging is the greatest risk factor for the development of PD [53, 54], dysregulation of cav-1 by parkin inactivation may accelerate senescence, causing neurodegeneration. Interestingly, overexpression of α-synuclein, a PD-associated gene product, also upregulates cav-1 expression, which mediates α-synuclein neurotoxicity [55, 56], supporting the involvement of the dysregulation of cav-1 in the pathogenesis of PD.

Cav-1 is well-known to play a role in the transport of cholesterol from the ER to the plasma membrane caveolae [28]. We demonstrated that accumulation of cav-1 by the loss of parkin increased the total cellular cholesterol level and decreased membrane fluidity. Upon cav-1 overexpression, total lipid cholesterol has been reported to be increased [57], which is in agreement with our study. Dysregulation of cholesterol metabolism has been largely reported to be associated with many neurodegenerative diseases including AD, PD and Huntington’s disease (HD) [58]. In a model of HD, mutant htt-expressing neurons exhibited cholesterol accumulation [59] and increased cav-1 expression. Loss or reduction of cav-1 expression in a mouse model of HD suppresses the motor phenotype [60], suggesting that cav-1 is a critical factor for neurodegeneration and could be a novel therapeutic target for many neurodegenerative diseases including PD. Changes in membrane fluidity by a variety of factors, as well as the alteration of cholesterol content, may affect membrane signaling properties. It has been reported that lipid changes in AD lipid rafts increase membrane order and viscosity in these domains [61]. APP/PS1 double transgenic mice exhibit age-dependent lipid rafts changes towards higher viscosity [62]. In addition, lipid rafts in patients with PD are notably more viscous and liquid-ordered than age-matched controls, which may ultimately contribute to progressive neuronal dysfunction [39], suggesting that subtle changes in membrane fluidity as a result of parkin dysfunction may affect a variety of membrane signaling, accelerating neurodegeneration.

We also demonstrated that accumulation of cav-1 by loss of parkin increased lipid rafts-dependent endocytosis. Fallon et al. [13] reported that EGFR endocytosis is accelerated in parkin-deficient cells, however, there is no difference in transferrin endocytosis. In addition, EGFR signaling is reduced in parkin KO mouse brain, proposing that the interaction between parkin and Eps15 plays a role in EGFR trafficking and signaling [63]. Based on previous reports that EGFR endocytosis is lipid rafts-dependent [64], it is in agreement with our findings that parkin regulates lipid raft-dependent endocytosis. Moreover, considering previous reports that EGFR signaling is attenuated by cav-1 overexpression in senescent cells [47] and breast cancer [65], attenuated EGFR signaling shown in parkin KO mouse brain is speculated to be partially mediated by the accumulation of cav-1.

Recent studies have highlighted prion-like mechanisms of propagation of aggregation prone proteins including α-synuclein, tau, and polyglutamine protein, which are associated with many neurodegenerative diseases and are anticipated to provide great insight into the pathogenesis of neurodegenerative diseases [66, 67]. In addition, numerous studies indicate functional interactions of PD-associated gene products and their involvement in common pathogenic mechanisms causing PD. Parkin protects against the toxicity associated with α-synuclein [68–70]. Transgenic mice expressing a mutant parkin exhibit accumulation of proteinase K-resistant α-synuclein [71]. GBA1 gene depletion, a strong genetic risk factor for PD, has recently been reported to enhance cell-to-cell transmission of α-synuclein [72]. In the present study, we observed that loss or reduction of parkin facilitated cell-to-cell transmission of α-synuclein, speculating that dysfunction of parkin may be involved in prion-like propagation of α-synuclein. Interestingly, compared with enhancement of lipid rafts-dependent endocytosis assessed by LacCer in parkin KD neurons, enhancement of α-synuclein uptake was much higher in parkin KD neurons, indicating that other endocytic pathways may be involved in α-synuclein uptake into neurons, and that parkin may also regulate these pathways. Parkin expression is known to be necessary for Lewy body formation [11] and parkin-mediated inclusion body formation is viewed as part of the cellular defense mechanism rather than a detrimental event [73, 74]. Accordingly, increased α-synuclein uptake without inclusion body formation in neurons with parkin depletion may be more detrimental. However, it has also been reported that the absence of parkin has no impact on the onset or progression of the phenotype induced by overexpression of human A53T α-synuclein [75]. Currently, it has not been fully unveiled as to what extent prion-like propagation of α-synuclein contributes to the pathogenesis of PD. Nevertheless, the association between the dysfunction of parkin and prion-like propagation of α-synuclein needs to be re-evaluated.

In addition to involvement in the pathogenesis of PD, parkin has been reported to function in metabolism [76, 77] and cancer [78] through the regulation of a variety of processes including receptor trafficking and mitochondrial quality control. Cav-1 has been well-reported to be associated with cancer and metabolism [79]. Accordingly, the involvement of parkin in many physiological and pathological conditions such as metabolism and cancer may be mediated by cav-1 expression.

## Conclusions

We observed the accumulation of cav-1 in parkin KO MEF cells and parkin KD neurons and identified cav-1 as a novel substrate of parkin. Accumulation of cav-1 by parkin dysfunction altered the total cholesterol levels and membrane fluidity, causing alterations in lipid rafts-dependent endocytosis. In addition, parkin dysfunction accelerated propagation of α-synuclein into neighboring cells, which may contribute to the progression of PD. Accordingly, regulation of cav-1 may be a novel target to elucidate the pathogenesis of PD.

## Methods

### Antibodies and reagents

The following primary antibodies (Abs) were used: anti-caveoin-1 (BD bioscience, Franklin Lakes, NJ; Abcam, Cambridge, MA), anti-caveolin-2 (BD bioscience), anti-flotillin-1 (BD bioscience, Franklin Lakes), anti-flotillin-2 (BD bioscience, Franklin Lakes), anti-CD71 (transferrin receptor, Santa Cruz Biotechnology, Santa Cruz, CA), anti-parkin (Santa Cruz Biotechnology, Santa Cruz, CA), anti-flag (Sigma-Aldrich, St. Louis, MO) and biotin conjugated anti-CTxB (Sigma-Aldrich, St. Louis, MO), anti-myc (Sigma-Aldrich, St. Louis, MO) and anti-actin (Santa Cruz Biotechnology, Santa Cruz, CA). MG-132 and lactacystin were purchased from Sigma-Aldrich (St. Louis, MO). Cycloheximide was purchased from Calbiochem (Denvers, MA). Rhodamine-conjugated transferrin and BOIPY® FL C_5_-Lactosylceramide were purchased from Invitrogen/Molecular Probes (San Dieogo, CA).

### Cell culture and transfection

WT and parkin KO mouse embryonic fibroblast (MEF) cells, kindly provided by Dr. Youle RJ (National Institutes of Health, Bethesda), and human α-synuclein overexpressing SH-SY5Y cells [36, 37] were grown in Dulbecco’s modified Eagle’s medium (DMEM) supplemented with 10 % fetal bovine serum (FBS). Cells were transfected using lipofectamine 2000 or lipofectamine RNAiMax (Invitrogen, Carlsbad, CA) according to the manufacturer’s instruction. After 48 or 72 h of transfection, cells were used for Western blotting or imaging analysis. Primary cortical neurons were cultured from Sprague-Dawley rat embryos at embryonic day 18, and maintained in Neurobasal medium (Invitrogen, Carlsbad, CA) with L-glutamine and B-27 supplement (Invitrogen, Carlsbad, CA). All animal procedures used in the present study were conducted according to the guidelines established by the Ajou University School of Medicine Ethics Review Committee for Animal Experiments.

### Constructs

The plasmid for flag-WT parkin was kindly provided by Dr. Chung KC (Yonsei University, Seoul), and the plasmid for His-ubiquitin was kindly provided by Dr. Park TJ (Ajou University School of Medicine, Suwon). The plasmids for parkin point mutants (T240R and T415N) were constructed using the QuickChange site-directed mutagenesis kit (Stratagene, La Jolla, CA). The plasmid for cav-1-EGFP was constructed using PCR. All constructs were verified by DNA sequencing and prepared using the Endo-Free plasmid Maxi prep Kit (Qiagen, Valencia, CA) to remove endotoxin contamination. siRNA for murine cav-1 was purchased from Bioneer (Deajeon, Korea). Lentiviral shRNA was constructed using the shRNA plasmid for rat cav-1 (GeneCopoeia, Rockville, MD ), as described previously [80].

### Western blotting and immunoprecipitation

Cells were solubilized in lysis buffer containing 50 mM Tris-HCl, pH 7.4, 150 mM NaCl, 0.25 % sodium deoxycholate, 1 % Triton X-100, and protease inhibitor mixture *(*GenDEPOT, Barker, TX) for 30 min on ice. The lysates were cleared by centrifugation at 20,000 × g for 15 min at 4 °C. The supernatants were mixed with 5 × sample buffer, resolved by SDS-PAGE, transferred to PVDF membrane, and analyzed by immunoblotting using the indicated antibodies. For immunoprecipitation, precleared supernatants were incubated with antibody-bound protein G beads (Millipore, Billerica, MA) for 6 h at 4 °C and washed four times with lysis buffer. Immunoprecipitates were subjected to Western blotting.

### Isolation of the lipid rafts fraction

Cells were washed twice with ice-cold PBS and lysed in ice-cold PBS containing 1 % Triton X-100 and protease inhibitor mixture. Following incubation for 20 min at 4 °C, the lysates were centrifuged at 20,000 × g for 15 min at 4 °C. Supernatants were used as soluble fractions. The pellets were washed with ice-cold PBS buffer, solubilized with 1 X sample buffer and used as insoluble fractions. These individual fractions were analyzed by SDS-PAGE and Western blotting.

### Quantitative real-time PCR

Total RNA was extracted from cells using Trizol reagent (Invitrogen, Carlsbad, CA) and cDNA was prepared using avian myeloblastosis virus RT (Promega, Fitchburg, WI) according to the manufacturer’s instruction. cDNA samples were analyzed by the Rotor-Gene SYBR Green PCR Master mix kit on Rotor-Gene cyclers (QIAGEN, Hilden, Germany). The following primers were used: murine caveolin-1 forward, 5′- CGT AGA CTC CGA GGG ACA TC-3′ and reverse, 5′- TCC CTT CTG GTT CTG CAA TC -3′; murine caveolin-2; forward, 5′- CTC AAG CTA GGC TTC GAG GA-3′ and reverse, 5′- GCA AGA CCA TTA GGC AGG TC - 3′; murine flotilin-1; forward, 5′- AGA AGC CTT CCA GAT GTA CC-3′ and reverse, 5′- ATG TCC AGT ACT TCC CCA GT - 3′; murine flotilin-2; forward, 5′- TAA GGC TGA GGC CTA CCA GA -3′ and reverse, 5′- AGC AGC CGG TTT ACT TCT GA - 3′; murine actin; forward, 5′- TGT TAC CAA CTG GGA CGA CA - 3′ and reverse, 5′-GGG GTG TTG AAG GTC TCA AA - 3′. Gene expression was normalized to the endogenous housekeeping control gene, β-actin, which was not influenced by parkin deficiency. Relative expression was calculated for each gene using the Δ Δ C_T_ (where C_T_ is the threshold cycle) method. Statistical analysis of reverse-transcription-PCR (RT-PCR) data was based on duplicate samples.

### *In situ* proximity ligations assay (PLA)

The PLA was performed using the DuoLink PLA kit (Sigma-Aldrich, St. Louis, MO, USA) according to the manufacturer’s instructions. Briefly, cells were fixed with 4 % paraformaldehyde and permeabilized with 0.1 % Triton X-100. Following treatment with DuoLink blocking buffer, cells were incubated with diluted primary antibodies against parkin and cav-1. After washing, cells were incubated with species-specific PLA probes and two additional oligonucleotides under hybridization conditions. Hybridization occurs when PLA probes are in close proximity, which can be subsequently ligated to form a closed circle. A rolling-circle amplification step follows with polymerase to generate a concatemeric product, which can be visualized with fluorephore-labeled oligonucleotides after hybridization. The slides were stained with DAPI and analyzed using confocal microscopy (Zeiss, Germany).

### Measurement of membrane fluidity using C-laurdan

MEF cells and primary cortical neurons cultured on poly-D-lysine coated glass were incubated with cell culture media containing 0.5 μM C-laurdan (SFP Co., Ltd, Chungbuk, Korea) for 20 min at 37 °C. The membrane fluidity was observed using two-photon fluorescence images, which were obtained with spectral confocal multiphoton microscopes (Leica TCS SP2, Wetzlar, Germany). The intensity images of C-laurdan were recorded with emissions in the range of 400–460 nm and 470–530 nm, with two channels of PMTs. Generalized polarization (GP) values for each pixel were obtained using the GP analysis program, an ImageJ plugin program and were processed as described previously [30]. GP values ranged from -1, corresponding to the highest fluidity, to +1 for the lowest fluidity.

### Total cellular lipid extract and quantification of cholesterol

Total cellular lipid was extracted using the Bligh-Dyer method [81]. Briefly, 3 ml chloroform:methanol (2:1,v/v) was added to 1 ml medium containing 10^6^ cells, followed by vigorous vortexing for 15 min. One ml of 1 M NaCl was then added to the solution and vortexed for several seconds, and the mixture was centrifuged at low speed to separate the two phases (2,000 rpm for 15 min at room temperature). The upper phase was removed by a glass pipette and discarded. The interface was rinsed once with methanol:water (1:1) without mixing the whole preparation. The lower chloroform phase, containing lipids, was carefully transferred to fresh tubes and evaporated under N_2_ gas. The lipid residue was resuspended in phosphate buffer containing 0.1 M potassium phosphate, pH 7.4, 50 mM NaCl, 5 mM cholic acid, and 0.1 % Triton X-100. Total cholesterol levels were quantified using an Amplex Red cholesterol assay (Life Technologies, Carsbad, CA) according to the manufacturer’s instruction.

### Confocal microscopy

Cells cultured on poly-D-lysine coated coverslips were washed twice with PBS and fixed in 4 % paraformaldehyde for 30 min at room temperature. The fixed cells were then washed with PBS and incubated with PBS containing 0.1 % Triton X-100 for 5 min at room temperature. After washing several times with PBS, cells were blocked with PBS containing 1 % bovine serum albumin (BSA) and 0.1 % Triton X-100 for 30 min at room temperature, and then incubated overnight with primary antibodies at 4 °C. Preparations were then stained with fluorescence-conjugated secondary antibodies for 2 h, mounted with vectashield (Vector Laboratories, Burlingame, CA), and observed under a confocal microscope (Zeiss, Germany).

### Statistical analysis

All values are expressed as the mean ± SEM. Statistical significance was evaluated using the Graphpad software (San Diego, CA).
